# Metabolic state and female fertility in ART cycles: a summary of current advances

**DOI:** 10.3389/fcell.2025.1645127

**Published:** 2025-09-23

**Authors:** Natascha Berger, Katharina Brugger, Herbert Fluhr

**Affiliations:** Department of Obstetrics & Gynecology, Medical University of Graz, Graz, Austria

**Keywords:** infertility, metabolism, reproduction, IVF, biomarker

## Abstract

This mini review explores the evolving landscape of metabolic disturbances and their impact on female reproductive outcomes, with a particular focus on emerging molecular biomarkers and novel metabolic phenotypes. Metabolic health plays a pivotal role in female reproductive function, with well-established endocrine-metabolic disorders, such as polycystic ovary syndrome, obesity, and diabetes mellitus, known to impair fertility. This review explores these conditions, including less commonly studied phenotypes like normal weight obesity, metabolically obese normal weight, and metabolically healthy obesity, which challenge traditional diagnostic frameworks by presenting metabolic risk independent of body mass index. These underrecognized profiles can adversely affect ovarian physiology, endometrial receptivity, and assisted reproductive technology outcomes. The review further highlights potential biomarkers, including bile acids and advanced glycation end-products, as promising diagnostic and therapeutic targets. By integrating insights from metabolic regulation, endocrinology, and reproductive biology, this article emphasizes the need for a more nuanced, individualized approach to fertility assessment and treatment. Understanding these interconnections is vital for advancing personalized medicine, particularly in addressing unexplained infertility and optimizing assisted reproductive technology success.

## Introduction

Infertility affects 8%–12% of women of reproductive age (Chambers et al., 2021) and is a growing global concern, underscored by over 1 million assisted reproductive technologies (ART) cycles reported in 40 European countries in 2019 (Smeenk et al., 2023). Despite ongoing efforts, ART success rates remain low at around 30% (Smeenk et al., 2023), largely due to the complex and multifactorial nature of infertility etiologies. Recent evidence highlights that metabolic disorders, marked by insulin resistance, low-grade systemic inflammation, abdominal obesity, adipose tissue dysfunction and dyslipidemia, commonly observed in obese women and women with polycystic ovary syndrome (PCOS), are directly implicated in reduced fertility (Broughton and Moley, 2017; Helvaci and Yildiz, 2024; Zhuang et al., 2024). Obesity and overweight are intricately linked to diminished pregnancy rates, heightened demands for gonadotrophins, and elevated occurrences of miscarriage (Dornelles et al., 2022; Hu et al., 2022; Qi et al., 2022). Moreover, a high BMI correlates with adverse pregnancy outcomes, including gestational diabetes, hypertension, and preterm labor (Madan et al., 2010; Šimják et al., 2018; Liu et al., 2019). The underlying mechanisms are versatile and encompass hormonal alterations due to hypothalamic-pituitary-ovarian axis disruption, derailed metabolism (insulin resistance, hyperglycemia, dyslipidemia), chronic inflammation (elevated levels of proinflammatory cytokines, e.g., IL-6, IL-1β, TNF-α) and mitochondrial dysfunction (enhanced oxidative stress, apoptosis, mitochondrial DNA damage) that ultimately induce alterations in the ovarian follicle microenvironment, that have been extensively reviewed elsewhere (Silvestris et al., 2018; Gonnella et al., 2022; Muhammad et al., 2023). Alarmingly, the World Obesity Federation highlights a striking uptick in the prevalence of maternal obesity within developed nations, which is projected to reach 45%–50% by 2030 (Phelps et al., 2024). With obesity and related metabolic disorders on the rise, assessing metabolic risk factors is essential to optimize infertility treatments and personalize strategies for improving pre-conception health. This article reviews current evidence on how metabolic health influences female fertility, highlighting emerging perspectives and under-studied phenotypes, normal weight obesity (NWO), metabolically obese normal weight (MONW), and metabolically healthy obesity (MHO), in relation to reproductive outcomes. Additionally, it explores the potential of emerging molecules such as bile acids (BAs) and advanced glycation end-products (AGEs) as biomarker candidates, and concludes with an overview of current research gaps and future directions.

## Emerging metabolic phenotypes and female fertility

Based on the World Health Organization guidelines, a body mass index (BMI) equal to or exceeding 25 kg/m^2^ stratifies overweight and a BMI above 30 kg/m^2^ obesity, respectively (Lobstein et al., 2023). The use of BMI remains the foremost widely accepted measure for evaluating overweight and obesity. However, the diagnostic utility of BMI is significantly limited due to its inability to differentiate between fat and lean mass, which may lead to misclassifications since individuals sharing the same BMI may exhibit markedly distinct fat distributions. Women tend to face a higher likelihood of being underrecognized for overweight or obesity based on BMI criteria alone (Peterson et al., 2014). This lack of discrimination may hinder accurate assessments of adiposity associated health risks and necessitates the integration of additional measures, such as waist circumference or percentage of body fat (%BF). Emerging data indicates that central adiposity (higher waist-hip ratio) predicts lower fecundability and live birth after in-vitro fertilization/intracytoplasmic sperm injection (IVF/ICSI) independent of BMI (Li et al., 2019; Ye et al., 2025). Notably, recent evidence suggests that the negative impact of central obesity is even more pronounced in women with lower BMI (Ye et al., 2025). Based on the data of the National Health and Nutrition Examination Survey, threshold values for %BF were established by evaluating its correlation with the risk of developing metabolic syndrome at various BMI cut-off points. For women, the %BF thresholds were defined at 24%, 31%, 37%, and 43%, which correspond to BMI values of 18.5, 25, 30, and 35 kg/m^2^, respectively (Zhu et al., 2003). In this context, studies have highlighted the importance of differentiating individuals with NWO from those who are MONW. NWO is characterized by a BMI in the normal range (18.5–24.9 kg/m2) but elevated %BF (Mohammadian Khonsari et al., 2022). MONW individuals present a normal BMI but high %BF and obesity-related metabolic disturbances, such as excess visceral fat, insulin resistance, dyslipidemia, hypertension, and elevated risk of type 2 diabetes mellitus (T2D) and cardiovascular events (Pluta et al., 2022). In NWO individuals, metabolic abnormalities are often subtler and frequently undetected, however, this phenotype is associated with an elevated risk of metabolic syndrome and cardiometabolic disease compared with individuals of normal BMI and %BF (Table 1) (Mohammadian Khonsari et al., 2022). A cross-sectional study in Korea found that men and women with NWO were 2.7 and 1.9 times more likely to develop metabolic syndrome, respectively, compared to those with a normal BMI and %BF (Kim et al., 2023). A study comparing 326 males and 641 females revealed that 26% of males and 38% of females within the normal BMI range had a %BF above the respective cutoffs of 25% and 35% (Lahav et al., 2023). Furthermore, they showed that even a slightly elevated waist circumference (>88 cm in women) can identify 60% of NWO individuals at risk for metabolic syndrome (Lahav et al., 2023). A recent study on NWO found that 15.8% of 469 normal-weight women had elevated %BF (≥31%), which was associated with reduced reproductive outcomes, including lower antral follicle counts, numbers of retrieved oocytes, fertilized oocytes, high-quality embryos on day 3 post fertilization and cleaved embryos (Yao et al., 2024). Interestingly, these findings stand in contrast to previous research, which failed to identify disparities in antral follicle counts or the quantity of retrieved oocytes between women categorized by %BF status. (Kim et al., 2021). Both studies found no differences in early pregnancy outcomes, including implantation, biochemical pregnancy, and clinical pregnancy rates, when women were stratified by %BF (Kim et al., 2021; Yao et al., 2024). In a large BMI-stratified IVF cohort, MONW had lower biochemical pregnancy rates than metabolically healthy normal-weight counterparts, with high blood pressure identified as a significant risk factor for this outcome (Ding et al., 2025). However, variations in cardiometabolic risk factors within the same BMI category appeared to have limited impact on live-birth rates (Ding et al., 2025).

**TABLE 1:** Criteria for defining NWO, MONW and MHO phenotypes.

Phenotype	Characterization	Increased risk of^a^
NWO (Mohammadian Khonsari et al., 2022)	BMI 18.5–24.9 kg/m2 BF% >32 Central obesity	hyperglycemia, insulin resistance, hypertension, dyslipidemia, metabolic syndrome
MONW (Ruderman et al., 1998; Pluta et al., 2022)	BMI 18.5–24.9 kg/m2 BF% ≥32 Excess visceral fat, hyperinsulinemia and insulin resistance, dyslipidemia, PCOS	metabolic syndrome, T2D cardiovascular events
MHO (Smith et al., 2019)	BMI ≥25 kg/m2, <35 kg/m2 Absence of metabolic abnormalities or diagnosis ≤2 of 5 metabolic syndrome components	Cardiometabolic disturbances

^a^ compared to metabolically healthy normal weight individuals.

Importantly, the prevalence of NWO and MONW in women varies with age, showing that advancing age and concomitant menopause leads to an increased risk of metabolic abnormalities (Zheng et al., 2020; Lahav et al., 2023). One explanation is that estrogen may act as a protective factor in maintaining metabolic health in normal-weight females, since the loss of estrogen signaling in menopausal women leads to a preferential increase in visceral fat (Hetemäki et al., 2021).

Of further specific interest are obese individuals demonstrating minimal or no metabolic disturbances, a condition termed MHO (Table 1) (Smith et al., 2019). Large-scale genomic studies have identified genetic variants associated with favorable cardiometabolic profiles despite increased adiposity, often located in or near genes involved in adipogenesis, fat distribution and insulin signaling, suggesting a genetic basis for this phenotype (Huang et al., 2018). A recent study of women aged 20–45 found that both metabolically healthy and unhealthy individuals with obesity had similar infertility risk, suggesting obesity itself is a key factor regardless of metabolic status. However, metabolically unhealthy obese individuals exhibited a significantly pronounced risk of infertility compared to MHO individuals (Tang et al., 2023). Similarly, the S-PRESTO preconception cohort found reduced fecundability associated with metabolic disease, predominantly in overweight and obese women, whereas no reduction was observed in those with metabolically healthy profiles by either metabolic syndrome criteria or HOMA-IR (Loy et al., 2022). Complementary plasma proteome analysis revealed that reproductive system development pathways were significantly downregulated in MHO individuals compared to lean healthy controls (Mir et al., 2025). In summary the current body of literature indicates that obesity, irrespective of metabolic health, is associated with impaired fertility, suggesting that weight management should be a priority in reproductive health strategies, even among metabolically healthy individuals. While there is extensive research on the impact of obesity on fertility, the specific effects of NWO, MONW and MHO on fertility, *in vitro* embryo development and pregnancy outcomes remain areas that require further investigation.

## Metabolic disorders and metabolite profiles in female infertility

PCOS is a common endocrine disorder affecting 4%–21% of women of reproductive age and is a leading cause of anovulatory infertility (Lizneva et al., 2016). Obesity, present in 50%–80% of PCOS cases, plays a central role in the pathophysiology of PCOS by promoting insulin resistance, which affects approximately 44%–70% of affected women (Sam, 2007; Diamanti-Kandarakis and Dunaif, 2012). However, the interplay between obesity and PCOS is complex and appears to be bidirectional: it remains unclear whether PCOS predisposes women to weight gain or if obesity itself initiates the metabolic disturbances that culminate in PCOS. In fact, both obese and non-obese women with PCOS exhibit more adverse metabolic profiles and increased visceral adiposity compared to age- and BMI-matched controls. Non-obese individuals often display features of the MONW phenotype, including hyperinsulinemia, insulin resistance, and elevated intra-abdominal fat mass (Ruderman et al., 1998; Satyaraddi et al., 2019). Moreover, insulin resistance is present in approximately 80% of obese PCOS patients and even in 20% of their nonobese counterparts, underscoring that significant metabolic risk persists regardless of BMI (Satyaraddi et al., 2019). Similarly, women with diabetes encounter reproductive health issues including a higher prevalence of oligomenorrhea, irregular menses, subfertility, adverse pregnancy outcomes, and early menopause (Thong et al., 2020; Qin et al., 2023). Since these reproductive issues are partially attributed to concomitant obesity and PCOS, studies exclusively discerning the impact of diabetes are of great importance. Women with T2D may experience equal or greater fertility risks than those with type 1 diabetes mellitus (T1D), despite a shorter disease duration (Thong et al., 2020; Mattsson et al., 2021). Aberrant insulin actions and hyperglycemia are significant drivers that contribute to ovarian dysfunction (Dupont and Scaramuzzi, 2016). The ovary expresses insulin receptors in granulosa cells, theca cells, and stromal tissue. Insulin signaling, through insulin receptors and insulin-like growth factor-1 receptors, can mimic follicle-stimulating hormone (FSH) and luteinizing hormone (LH) actions, a phenomenon known as the co-gonadotropin effect, enhancing androgen, estrogen, and progesterone production (Dupont and Scaramuzzi, 2016). By mimicking gonadotropins, insulin drives early follicular growth and contributes to polycystic ovarian morphology (Kwintkiewicz and Giudice, 2009). Furthermore, in human granulosa cells, insulin promotes free fatty acid synthesis and synergizes with luteinizing hormone to upregulate key sterol-regulatory genes (Sekar et al., 2000; Richardson et al., 2005). In obese PCOS patients with insulin resistance, follicular fluid contains significantly elevated levels of mono- and polyunsaturated fatty acids, which are linked to reduced oocyte quality and increased embryo fragmentation (Niu et al., 2014). Supporting these findings, PCOS granulosa cells exhibit dysregulated expression of genes involved in lipid metabolism, fatty acid biosynthesis, and steroidogenesis, including upregulation of lipoxygenases that promote pro-inflammatory lipid mediator production (Li et al., 2017; Liao et al., 2022).

As key metabolic regulators of glucose and lipid homeostasis and modulators of systemic inflammatory balance bile acids (BAs) have emerged as crucial biomolecules in PCOS pathophysiology (Fleishman and Kumar, 2024). Since abnormal circulating BA profiles are associated with metabolic disorders, including obesity and T2D (Li and Chiang, 2014; Qi et al., 2021), and complications and diseases during pregnancy (Brouwers et al., 2015), there is a growing interest in the underlying molecular mechanisms and the establishment of BAs as biological markers to determine pathological changes. In PCOS patients, an elevation of distinct BA classes and direct associations of BA species with insulin and androgen levels were demonstrated (Zhang et al., 2019; Yu et al., 2023). Moreover, shot-gun sequencing of stool samples in PCOS identified a gut microbiota imbalance linked to reduced glycine- and taurine-conjugated BA species (Qi et al., 2019). Fecal microbiota transplantation from PCOS patients induced ovarian dysfunction, insulin resistance, disrupted BA metabolism and infertility, alongside reduced interleukin-22 secretion in mice. BA treatment stimulated IL-22 production and ameliorated PCOS features, highlighting gut microbiota, BA metabolism and IL-22 modulation as potential therapeutic targets (Qi et al., 2019).

Chronic hyperglycemia further promotes the formation of advanced glycation end-products (AGEs). AGEs are heterogeneous and nondegradable molecules formed endogenously or ingested through diet, via non-enzymatic reactions between reducing sugars and free amino groups of proteins, lipids, or nucleic acids, disrupting the normal function of these molecules. Accumulation of AGEs induces tissue damage through receptor-independent binding to the extracellular matrix or receptor-dependent activation of receptor for advanced glycation end-products (RAGE), both of which trigger inflammatory and oxidative stress responses (Mengstie et al., 2022). Additionally, the soluble receptor for AGEs (sRAGE) circulates systemically and acts as a decoy, mitigating AGE-induced damage by preventing their interaction with RAGE (Merhi, 2014). In follicular fluid, sRAGE protein levels were directly associated with the number of oocytes, follicles, ovarian sensitivity index, embryonic development, and successful clinical pregnancy outcomes in women undergoing ART, proposing sRAGE levels as a predictor for ART outcomes (Merhi et al., 2014; Nejabati et al., 2017; Li et al., 2017). Accordingly, the presence of AGEs in serum and follicular fluid of women undergoing ART has been significantly correlated with poor follicular and embryonic development, as well as a reduced likelihood of achieving an ongoing pregnancy (Jinno et al., 2011). Further, overweight and obese women have significantly lower serum sRAGE concentrations when compared with normal-weight women undergoing ART (Merhi et al., 2014). This finding is in line with previous studies demonstrating low plasma sRAGE levels in obese women and diabetic patients, which are associated with high BMI, blood pressure-, triglyceride-, HbA1c-levels, an increased insulin resistance index and enhanced thromboxane biosynthesis, potentially contributing to obesity-related metabolic and vascular disease (Koyama et al., 2005; Vazzana et al., 2012). An *in vitro* study examining the impact of AGEs on insulin signaling and glucose transport using a human ovarian granulosa cell line demonstrated that glycated albumin inhibits PI3K-specific AKT phosphorylation and prevents Glut-4 translocation to the cell membrane, indicating that intra-follicular accumulation of AGEs may interfere with physiological functions of granulosa cells contributing to impaired follicular development (Diamanti-Kandarakis et al., 2016). Immunohistochemical analysis has shown elevated levels of AGEs and RAGE in granulosa and theca cells of women with PCOS and demonstrated a direct association between hyperandrogenism and AGEs in the condition (Garg and Merhi, 2016; Azhary et al., 2020). Of note, administration of the hydrophilic tertiary BA tauroursodeoxycholic acid, a clinically used endoplasmic reticulum stress inhibitor, significantly reduced RAGE and AGE expression in granulosa cells and decreased the number of atretic follicles in PCOS mice (Azhary et al., 2020; Yuan et al., 2024).

## Discussion

The presence of hormonal and metabolic derailments as seen in PCOS, diabetes, NWO, MONW, and MHO underscores the multifaceted nature of infertility etiologies. Unexplained infertility in approximately 15% of couples further emphasizes the need to investigate less apparent disease patterns and delve into molecular pathways influencing female fertility (Romualdi et al., 2023). Further investigation of NWO, MONW, and MHO phenotypes in reproductive biology is essential, as limited research exists and these conditions are often overlooked, underscoring the need to incorporate comprehensive metabolic assessments into routine clinical care. Women exhibiting a normal BMI are frequently underdiagnosed as studies have shown that approximately 40% of 641 screened women had a BF% above the recommended cutoffs (Lahav et al., 2023). Prospective epidemiological studies are essential to validate these findings and examine the long-term impact of NWO, MONW, and MHO on female fertility.

PCOS diagnosis involves multiple markers, including androgen levels and insulin resistance indicators, to characterize the syndrome’s phenotype. Women with PCOS also display adverse lipid profiles and elevated cardiovascular risk, emphasizing the need for targeted metabolic interventions. In this context, BAs have emerged as potential diagnostic biomarkers and novel systemic signaling molecules (Vítek and Haluzík, 2016; Fiorucci et al., 2021). Particularly pharmacological strategies to modulate the activity of the nuclear BA receptor Farnesoid X Receptor and the G-protein coupled BA receptor TGR5 holds promise for treating fatty liver diseases, obesity, diabetes, and related disorders (Li and Chiang, 2014). Importantly, the composition of BAs as well as the level of total BAs in follicular fluid have been reported to affect follicular maturation and steroidogenesis in different animal models (Takae et al., 2019; Wei et al., 2022; Zhu et al., 2025). Although promising preclinical evidence supports a significant impact of BAs on the intrafollicular milieu in PCOS, more detailed basic research utilizing human *in-vitro* models are necessary to establish the efficacy of specific BA-targeting interventions.

Moreover, chronic hyperglycemia, oxidative stress, and chronic low-grade inflammation are key factors known to drive the endogenous formation of AGEs. These compounds have been implicated in the pathogenesis and progression of numerous chronic diseases, including T2D, cardiovascular disease, Alzheimer’s disease, and inflammatory bowel diseases (Perrone et al., 2020; Mengstie et al., 2022). The potential of anti-AGE strategies, including agents such as metformin, to alleviate AGE-related dysfunctions in PCOS has been demonstrated (Merhi et al., 2019). Furthermore, treatment with tauroursodeoxycholic acid, has shown promise in reducing AGE and RAGE expression and improving ovarian health in PCOS models (Azhary et al., 2020). The mechanisms through which anti-AGE interventions may exert their effects include reducing oxidative stress, improving insulin sensitivity, and modulating inflammatory pathways, which are critical in the management of PCOS. Understanding the AGE-RAGE/sRAGE axis in ovarian dysfunction, especially in women with PCOS, may enhance our understanding of pathologic female reproductive mechanisms. Despite the critical role of AGEs in various disease mechanisms, there is currently no standardized detection method for these compounds, nor is there routine monitoring in clinical practice. Advancing our understanding of AGEs' formation and their pathological impacts could pave the way for improved diagnostic and therapeutic strategies.

## Research gaps

### Beyond BMI—better phenotyping

Given BMI’s limitations as a proxy for adiposity and metabolic disease risk, adjunct anthropometrics (e.g., waist-to-hip ratio) and body-composition measures (e.g., %BF) may offer clinically useful refinement. Further, rigid BMI-based criteria for offering or denying IVF may restrict equitable access without evidence of clinical benefit. Thresholds (often ≥35–40 kg/m^2^) vary widely across programs and are largely grounded in observational associations between higher BMI, lower ART success, and greater obstetric risk rather than randomized evidence (Penzias et al., 2021).

### Insulin resistance—Standardize and stratify

Insulin resistance, prevalent in PCOS and obesity-related infertility, is inconsistently defined in the literature, with most studies using surrogate indices (e.g., HOMA-IR, QUICKI, Belfiore’s index) that vary in accuracy and underperform the euglycemic–hyperinsulinemic clamp, particularly in lean women (Gayoso-Diz et al., 2013; Biernacka-Bartnik et al., 2023). This heterogeneity limits comparability, obscures prevalence in ART populations, and hampers reproducibility of outcome associations. Standardized, cardiometabolic-risk–based and age-adjusted definitions, potentially integrating biomarkers such as insulin growth factor binding protein, adiponectin and ferritin combined with surrogate indices (Martínez-García et al., 2009; Brismar et al., 2023), could improve diagnostic precision and guide metabolic optimization in ART.

### Lifestyle, weight-loss and pharmacotherapy interventions before ART

Major societies (European Society of Human Reproduction and Embryology (ESHRE) and the American Society for Reproductive Medicine (ASRM)) recommend lifestyle interventions for individuals with overweight/obesity to reduce weight, central adiposity, and insulin resistance. However, randomized controlled trails and meta-analyses indicate that pre-IVF weight-loss programs (diet/exercise ± medication) improve interim outcomes (e.g., unassisted conception) but have inconsistent or no effect on live birth for women proceeding to IVF (Penzias et al., 2021; Jeong et al., 2024). Pre-ART metabolic pharmacotherapies (metformin, anti-obesity pharmacotherapy including GLP-1 receptor agonists) are supported by small, heterogeneous studies that suggest potential benefit by emphasizing surrogate endpoints (oocyte yield, biochemical pregnancy) but require confirmation in large randomized controlled trails powered for live birth and safety (Kotlyar and Seifer, 2023).

## Future directions

Next-generation personalization in reproductive medicine will likely come from integrating multi-omics with clinical workflows, potentially improving non-invasive prediction of oocyte competence and embryo viability. Metabolomics of follicular fluid, serum, and embryo culture media can yield dynamic snapshots of energy metabolism, oxidative balance and profile metabolites acting as crucial bioactive signaling molecules (Hood et al., 2022; Alizadeh Moghadam Masouleh et al., 2025; Zhang et al., 2025). Standardized sampling/analytics and prospective validation, ideally supported by machine-learning, are essential to develop reliable tools that link metabolic profiles with clinical outcomes.

Further, the gut microbiota is a central regulator of female reproductive health. The bidirectional “gut–reproductive axis,” mediated by interconnected immune, metabolic, and neuroendocrine networks, facilitates communication between the intestinal microbiota and reproductive organs (Moustakli et al., 2025). Dysbiosis, marked by altered short-chain fatty acid profiles, disrupted BA metabolism, and impaired gut barrier integrity, can perturb host energy balance, promote chronic inflammation, and contribute to metabolic disorders such as PCOS and obesity (Qi et al., 2019; Yao et al., 2023; Moustakli et al., 2025). Strategies to restore a healthy gut microbial profile, via dietary modification, prebiotics, probiotics, or targeted microbial therapeutics, hold promise for improving metabolic homeostasis and reproductive outcomes, though robust interventional evidence remains limited. Future research should focus on robust studies establishing causal relationships between the intestinal microbiome and reproductive disorders.

**FIGURE 1:**
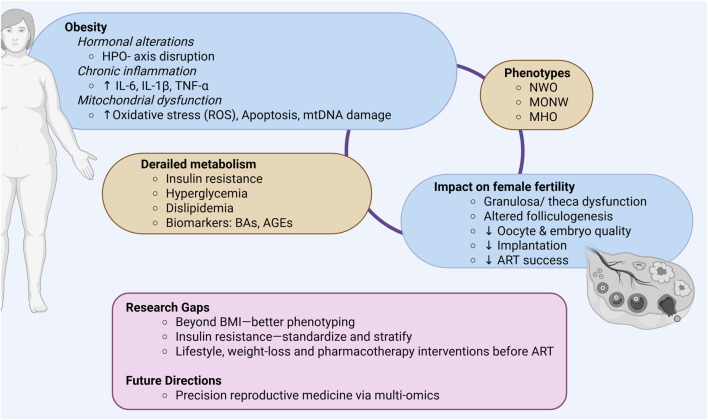
Graphical summary of key points discussed. HPO- axis, Hypothalamic-pituitary-ovarian- axis; IL-6, Interleukin-6; IL-1β, Interleukin- 1beta; TNF-α, Tumor necrosis factor alpha; ROS, Reactive oxygen species; mtDNA, Mitochondrial DNA; NWO, Normal weight obesity; MONW, Metabolically obese normal weight; MHO, Metabolically healthy obesity; BAs, Bile acids; AGEs, Advanced glycation end-products; ART, Assisted reproductive technologies; Created in BioRender.

Lastly, pharmacogenetics represents an emerging avenue for personalization in ART, with significant potential to optimize drug selection and dosing in ovarian stimulation and ovulation induction. Variants in FSH and LH receptor genes have been shown to influence ovarian response to standard stimulation protocols, and early evidence indicates that genotype-guided gonadotropin selection may enhance cumulative pregnancy and live birth rates (Conforti et al., 2025; Hjelmér et al., 2025). These findings warrant confirmation in randomized controlled trials and may ultimately enable tailoring of stimulation regimens to each patient’s metabolic–hormonal profile (Figure 1).

## Abbreviations

AGE, Advanced glycation end-product; ART, Assisted reproductive technologies; BA, Bile acid; %BF, Body fat percentage; BMI, Body mass index; MHO, Metabolically healthy obesity; MONW, Metabolically obese normal weight; NWO, Normal weight obesity; PCOS, Polycystic ovarian syndrome; T1D, Type 1 diabetes mellitus; T2D, Type 2 diabetes mellitus; IVF/ICSI, In-vitro fertilization/intracytoplasmic sperm injection; RAGE, Receptor for advanced glycation end-products; FSH, Follicle-stimulating hormone; LH, Luteinizing hormone.
